# Veterinary Medical Association of New Jersey

**Published:** 1886-07

**Authors:** William Herbert Lowe

**Affiliations:** Secretary


### VETERINARY MEDICAL ASSOCIATION OF NEW JERSEY.

The second annual meeting (seventh regular) of the Veterinary Medical Association of New Jersey, was held at the

United States Hotel in Morristown, on Thursday, April 8th, 1886,

The President, Dr. Wm. B. E. Miller, of Camden, occupied the chair and called the meeting to order at 11 A.M., when the roll was called by the Secretary. The following members were present:

David J. Dixon, of Hoboken; L. R. Sattler, of Newark; W. P. Humphrey, of Elizabeth; James C. Dustan, of Morristown; Wm. B. Haydon, of Newark; A. S. Leatherman, of Clinton; Wm. B. E. Miller, of Camden; James W. Hawk, of Newark; Wm. P. Smith, of Trenton; Rudolph Leis, of Newark; L. P. Hurley, of Hopewell, and Wm. Herbert Lowe, of Paterson.

There were also present I. Newton Krowl, D.V.S., of Union Hill; Charles Kuhne, D.V.S., of Jersey City; Willet H. Cooper, V.S., of Salem, and others interested in the advancement of the veterinary profession.

The minutes of the last regular meeting, which was held at Camden, December 10th, 1885, was read by the Secretary and adopted.

The President, Dr. Miller, then delivered his annual address. He congratulated the association on the advancement made in the short time—only two years—since its organization, and especially for that made within the past year, since it became a corporate body. He said that we have now thirty-two active members, and that there are five applicants for admission. He paid a delicate tribute to the late A. P. Weeks, of Paterson, and the late Dr. William G. Schmidt, of Newark. Much stress was laid on the necessity and importance of members attending the meetings regularly; that they should lend their aid individually, as well as generally—that it is very important to obtain "some legislative action that may secure for us recognition, such as a profession so noble as ours is, so well deserving of. Let us use our influence to elect such legislators in the near future as will listen and consider well our appeal for the passage of such laws as will provide for us proper protection. The present status and outlook of the profession shows that there is ample field for well-qualified veterinary surgeons, and that there is now for them, and in the near future still more so, not only honorable recognition, but ample reward. The veterinarian has yet to play a very important part in our large cities, in the inspection of meat, milk, butter and other productions that are daily put upon our markets totally unfit for food. In the extermination and prevention of contagious diseases, in the sanitary work of local and State Boards of Health, so far as they relate to animals, and in some other respects the veterinarian must become better known as a sanitarian. The great money value of the higher breeds of horses, cattle, etc., now render his services indispensable, and of high value. "The call is for more and better educated men," who must be prepared by a thorough course of instruction in a reputable, legally chartered college.

The President also spoke of the present and future usefulness of the Association, and of the great advantage of combined over individual effort. In conclusion, he returned thanks for the honored bestowed and the confidence reposed in him.

He said: "To my assistant officers I return especial thanks for many courtesies and favors received at their hands, and for the faithful manner in which they each and all have performed their several duties. To Dr. Lowe, who was elected Secretary upon the resignation of Dr. Julius Gerth, I am especially obligated. In the discharge of his duties he has proven himself worthy the praises due a competent and faithful officer. It will be to me a life-long pleasure to refer with pride to the associations of the past two years, and they will be cherished with kindly recollections of you all while memory lasts."

The Secretary was reminded that he had omitted to state in the minutes of the last meeting that the names of members in arrears, not paid on or before the next meeting following, would have their names stricken from the roll.

On motion of Dr. Hawk, the Secretary was instructed to immediately notify all members one year or more in arrears, that their names will be stricken from the roll at the next meeting, to be held Aug. 12th, unless the Treasurer, meanwhile, receives the amount due the association.

Dr. Miller proposed the following amendment to the by-laws:

Art. 1, sec. 1. Any member of this association who shall refuse or neglect to pay his annual dues for a period of one year, shall be duly notified by the Secretary of his delinquincy within thirty days thereafter, and if the said member does not pay the same at or before the next regular tri-annual meeting, his name shall be dropped from the ro l.

Sec. 2. Any member who shall have been dropped for non-payment of dues, may, upon application made at any regular meeting, be re-instated to active membership on the payment of all back dues and assessments accrued from the time he was dropped, by a two-third vote of the members present at such regular meeting.

The Secretary presented the certificates of membership printed since the association was incorporated, all members receiving them being obliged to sign the new constitution and by-laws.

The following eight practitioners were admitted to membership: I. Newton Krowl, D.V.S., of Union Hill; Chas. Kuehne, D.V.S., of Jersey City; Eldon Loblin, D.V.S., of New Brunswick; Joseph Nayler, D.V.S., of Rahway; Willet H. Cooper, V.S., of Salem, B. F. King, V.S., of Little Silver; T. C. Sandford, V.S., of Asbury Park, and F. W. Hillyard, of Mount Holly.

The Board of Censors reported unfavorably on the application for membership of Andrew W. Axford, of Naughrightville, he not having complied with the requirements of the constitution and by-laws. William S. Stone, of Chatham, went before the Board of Censors, but failed to pass a satisfactory examination. Application for membership was made by John Kehoe, of Lyndhurst. The Board of Censors will act on his case at the next meeting, provided he appears for examination.

Dr. Lowe spoke at some length of the defects of the present U. S. Army Veterinary service, but owing to the pressure of business, resolutions which he wished to have passed were laid over to be considered at the next meeting.

Dr. Lowe read a letter from the eminent veterinarian, Dr. George Fleming, of England, but which did not bear upon the business of the society. These words: "Give my heartiest good wishes to your worthy President and to the members of your association," etc., led to remarks by Dr. Lowe on the life and writings of Dr. Fleming, which were very kindly and heartily received. In conclusion, Dr. Lowe proposed Dr. Fleming for honorary membership. He was unanimously elected with marked demonstrations of good will by the assemblage.

Dr. Hawk was of opinion that great vigilance should be used by the association in discovering irregular behavior on the part of any of its members or any practitioners outside of it, with the view of denouncing the same, so that in their every-day practice practitioners shall be obliged to pursue an honorable course towards each other, and those for whom they render service.

Dr. Hayden endorsed the views of Dr. Hawk.

Dr. Krowl did likewise, adding that had he not been highly pleased with the new code of ethics of the association, he would probably not be a member—that he would very much prefer to deal personally with the questionable behavior of some practitioners all alone, than to have the name of the assistance of the association, unless the code of ethics should be fully enforced.

Remarks were also made by Drs. Cooper and Miller, which, in the main, coincided with those already expressed.

Some members were of opinion that there should be uniform rates of charges for certain special kinds of service, but the idea generally prevailed that there were comparatively few which might not be so modified by circumstances as to render it impossible to be governed by rules applicable in all cases.

All the officers of last year were re-elected, with the exception of the Treasurer and Board of Trustees. The officers are: Dr. William B. E. Miller, of Camden, President; Dr. David J. Dixon, of Hoboken, 1st Vice-President; Dr. Charles K. Dyer, of Mount Holly, 2d Vice-President; Dr. William Herbert Lowe, of Paterson, Secretary; Dr. L. R. Sattler, of Newark, Treasurer; Board of Trustees—Dr. Willet H. Cooper, of Salem; Dr. John P. Smith, of Trenton; Dr. James W. Hawk, of Newark; Dr. A. S. Leatherman, of Clinton; Dr. I. Newton Krowl, of Union Hill.

The President appointed Drs. Sattler, Leatherman and Leis, delegates of the United States Veterinary Medical Association, also to the New York State Veterinary Society, and the Pennsylvania State Veterinary Medical Association.

Drs. Dixon and Krowl were appointed essayists for the next regular meeting.

An excellent dinner was served to the members of the Association at the hotel where the meeting was held. Several afterdinner speeches were made, but the most interesting was that by Dr. David J. Dixon, member of the New York State Veterinary Society, and 1st Vice-President of our Association. He entertained all present very happily by his remarks on both societies.

The next regular meeting will be held at Long Branch the second Thursday of August next.

WILLIAM HERBERT LOWE, D.V.S., *Secretary.*

